# [(2*S*,5*R*)-1-Methyl-5-phenyl­pyrrolidin-2-yl]diphenyl­methanol

**DOI:** 10.1107/S1600536811023403

**Published:** 2011-06-25

**Authors:** Julio Zukerman-Schpector, Angélica Venturini Moro, Diogo S. Lüdtke, Carlos Roque D. Correia, Edward R. T. Tiekink

**Affiliations:** a Departmento de Química, Universidade Federal de São Carlos, CP 676, 13565-905 São Carlos-SP, Brazil; b Instituto de Química, Universidade Estadual de Campinas, UNICAMP, CP 6154, CEP 13084-917 Campinas, Brazil; c Instituto de Química, Universidade Federal, de Rio Grande do Sul, UFRGS, Av. Bento Gonçalves 9500, 91501-570 Porto Alegre, RS, Brazil; d Department of Chemistry, University of Malaya, 50603 Kuala Lumpur, Malaysia

## Abstract

In the title compound, C_24_H_25_NO, the phenyl and diphenyl­methanol substituents are *syn* to each other. The pyrrolidine ring has an envelope conformation with the flap atom being the C atom bearing the phenyl substituent. The hy­droxy group forms an intra­molecular hydrogen bond with the pyrrolidine N atom, and the phenyl rings lie to same side of the mol­ecule. The crystal packing features C—H⋯π inter­actions. Two slightly displaced co-planar orientations were found for one of the phenyl rings; the major component had a site-occupancy factor of 0.782 (15).

## Related literature

For background to the highly enanti­oselective addition of aryl­zinc reagents to aldehydes, see: Yoon & Jacobsen (2003 ▶), Taylor, *et al.* (2011 ▶). For related structures, see: Moro *et al.* (2010 ▶); Shabbir *et al.* (2009 ▶). For details of the synthetic protocols, see: Walsh & Kozlowski (2008 ▶); Paixão, *et al.* (2008 ▶). For ring conformational analysis, see: Cremer & Pople (1975 ▶).
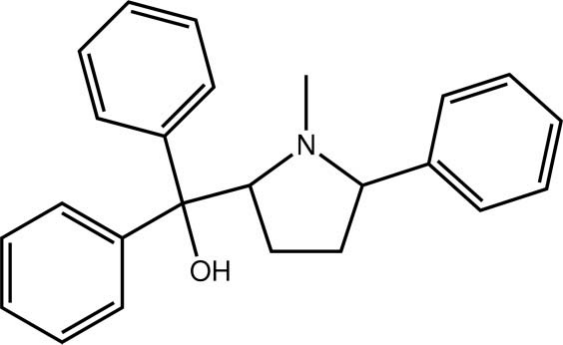


## Experimental

### 

#### Crystal data

C_24_H_25_NO
*M* *_r_* = 343.45
Orthorhombic, 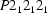

*a* = 9.9672 (2) Å
*b* = 13.3376 (2) Å
*c* = 14.4369 (2) Å
*V* = 1919.22 (5) Å^3^
*Z* = 4
Mo *K*α radiation
μ = 0.07 mm^−1^
*T* = 100 K
0.22 × 0.15 × 0.15 mm

#### Data collection

Bruker APEXII CCD diffractometer
24972 measured reflections
2262 independent reflections
1979 reflections with *I* > 2σ(*I*)
*R*_int_ = 0.039

#### Refinement

*R*[*F* ^2^ > 2σ(*F* ^2^)] = 0.038
*wR*(*F* ^2^) = 0.092
*S* = 1.05
2262 reflections
255 parameters
H-atom parameters constrained
Δρ_max_ = 0.17 e Å^−3^
Δρ_min_ = −0.19 e Å^−3^

### 

Data collection: *APEX2* (Bruker, 2007 ▶); cell refinement: *SAINT* (Bruker, 2007 ▶); data reduction: *SAINT*; program(s) used to solve structure: *SIR97* (Altomare *et al.*, 1999 ▶); program(s) used to refine structure: *SHELXL97* (Sheldrick, 2008 ▶); molecular graphics: *ORTEP-3* (Farrugia, 1997 ▶) and *DIAMOND* (Brandenburg, 2006 ▶); software used to prepare material for publication: *MarvinSketch* (Chemaxon, 2010 ▶) and *publCIF* (Westrip, 2010 ▶).

## Supplementary Material

Crystal structure: contains datablock(s) I, global. DOI: 10.1107/S1600536811023403/hg5054sup1.cif

Structure factors: contains datablock(s) I. DOI: 10.1107/S1600536811023403/hg5054Isup2.hkl

Additional supplementary materials:  crystallographic information; 3D view; checkCIF report

## Figures and Tables

**Table 1:** Hydrogen-bond geometry (Å, °) *Cg* is the centroid of the C6–C11 ring.

*D*—H⋯*A*	*D*—H	H⋯*A*	*D*⋯*A*	*D*—H⋯*A*
O—H1O⋯N	0.84	2.02	2.648 (2)	132
C28—H28⋯O^i^	0.95	2.78	3.359 (7)	120
C17—H17⋯*Cg*^ii^	0.95	2.92	3.776 (3)	150

Symmetry codes: (i) 
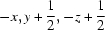
; (ii) 
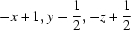
.

**Fig. 1.**
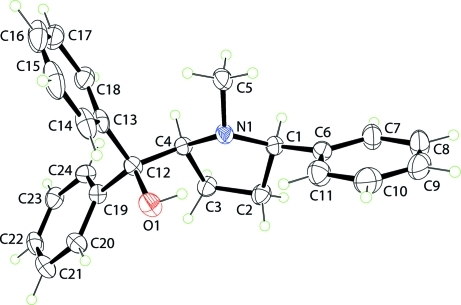
The molecular structure of compound (I) showing the atom-labelling scheme and displacement ellipsoids at the 35% probability level. Only the major component of the disordered benzene ring is illustrated.

**Fig. 2.**
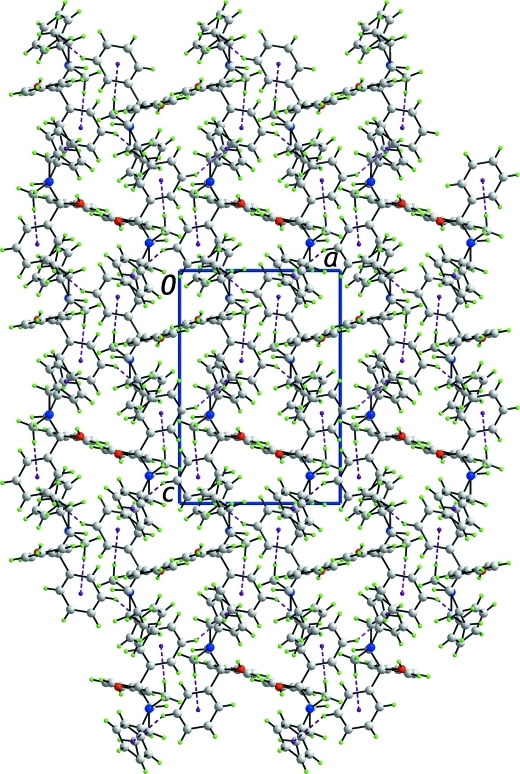
A view in projection down the b axis of the unit-cell contents for (I). The C—H···π interactions are shown as purple dashed lines.

## supplementary crystallographic information

### Comment

Chiral β-amino alcohols have found numerous applications in asymmetric catalysis in the past and continue to play a pivotal role in the development of new reactions and chiral ligands (Walsh & Kozlowski, 2008). One asymmetric reaction where chiral β-amino alcohol ligands have found enormous success is the enantioselective addition of organozinc reagents to carbonyl compounds, with particular emphasis in the alkylation of aldehydes by the addition of diethylzinc. A more challenging reaction is the asymmetric arylation reaction, since arylzinc reagents are more reactive than the dialkylzinc and the ligand turnover has to highly efficient in order to circumvent the uncatalyzed background reaction (Paixão *et al.*, 2008). Considering the proline motif as a privileged framework for the development of asymmetric catalysts (Yoon & Jacobsen, 2003) we have recently described a new chiral ligand for the highly enantioselective addition of arylzinc reagents to aldehydes. The ligands were prepared by an straightforward synthetic sequence, with a Heck reaction of arenediazonium salts (Heck–Matsuda reaction) as the key step (Taylor *et al.*, 2011). Herein, we describe the crystal structure analysis of a representative molecule, the title compound, (I).

The crystal structure analysis of (I) confirms the structure as having the expected *syn* relationship between the phenyl and the diphenylmethanol substituents, Fig. 1 (Moro *et al.*, 2010; Shabbir *et al.*, 2009). The pyrrolidine ring is in an envelope conformation with C1 out of the plane formed by the other four atoms, the ring puckering parameters being: q_2_ = 0.379 (2) ° and φ_2_ = 32.0 (3) ° (Cremer & Pople, 1975). The hydroxy group is orientated over the five-membered ring to facilitate the formation of an intramolecular O—H···N hydrogen bond, Table 1. The crystal packing is dominated by C—H···π interactions, Table 1. Globally. the pyrrolidine pack in the *ab* plane and are sandwiched by benzene rings along the *c* direction, Fig. 2.

### Experimental

The starting (2*S*)-1-*tert*-butyl 2-methyl 5-argio-1*H*-pyrrole-1,2(2*H*,5H)-dicarboxylate was prepared as described in previous work (Moro *et al.*, 2010). To a round-bottomed flask, under a hydrogen atmosphere, were added the Heck adduct ((2*S*))-1-*tert*-butyl 2-methyl 5-argio-1*H*-pyrrole-1,2(2*H*,5H)-dicarboxylate) (3 mmol) and dry methanol (60 ml), followed by the addition of Pd/C 10% (20% *w*/*w*, 0.18 g). The reaction was stirred at room temperature for 24 h. After this time, the crude reaction mixture was filtered in a plug of celite and concentrated under reduced pressure. The resulting product was used without further purification. To a round-bottomed flask, under an argon atmosphere, PhMgBr (5 equiv., 15 mmol) in THF (15 ml, 1 *M* solution) was added to a THF (10 ml) solution of the (2*S*)-1-*tert*-butyl 2-methyl 5-argiopyrrolidine-1,2-dicarboxylate (3 mmol) at room temperature, and the mixture was stirred for 4 h, before being quenched by careful addition of NaOH 2*M*. The heterogeneous mixture was filtered through a pad of Celite and washed with dichloromethane (3 *x* 50 ml). The combined organic phases were dried with MgSO_4_, filtered and the solvent removed under vacuum. The resulting product was used without further purification. To a suspension of lithium aluminium hydride (1.14 g, 30 mmol) in THF (15 ml) in a round-bottomed flask, under an argon atmosphere and cooled to 273 K, a solution of the (2*S*,5*R*)-*tert*-butyl 2-(hydroxydiphenylmethyl)-5-phenylpyrrolidine-1-carboxylate in THF (5 ml) was added. The resulting mixture was refluxed for 12 h. After this time, the mixture was cooled to 273 K and NaOH (4*M*) was added. The mixture was filtered through a pad of Celite and washed with ethyl acetate. The organic layer was separated, and the filtrate was extracted with ethyl acetate (3 *x* 50 ml). The combined organic phases were dried with MgSO_4_, filtered and the solvent removed under vacuum. The crude product was purified by flash chromatography in hexanes/ethyl acetate (95:05), to afford the 0.340 g (33%) of pure ((2*S*,5*R*)-1-methyl-5-phenylpyrrolidin-2-yl)diphenylmethanol (*cis* isomer) and 0.278 g (27%) of pure ((2*S*,5*S*)-1-methyl-5-phenylpyrrolidin-2-yl)diphenylmethanol (*trans* isomer) (60% combined yield). Suitable crystals for X-ray analysis were obtained by vapour diffusion from n-hexane/ethyl ether at 298 K. [α] _D_^20^ = +115 (c = 1.02, CHCl_3_). ^1^H NMR [CDCl_3_, 500 MHz, δ (p.p.m.)]: 1.66 (s, 3H, N—CH_3_), 1.68–1.83 (m, 2H, CH_2_), 1.92–2.03 (m, 2H, CH_2_), 3.54 (dd, J^1^ = 10.8 Hz, J^2^ = 6.0 Hz, 1H, CH), 3.89 (dd, J^1^ = 9.8 Hz, J^2^ = 4.0 Hz, 1H, CH), 4.97 (bs, 1H, OH), 7.09 (t, J = 7.0 Hz, 1H, Ar), 7.13 (t, J = 7.0 Hz, 1H, Ar), 7.19–7.33 (m, 9H, Ar), 7.58 (dd, J^1^ = 8.5 Hz, J^2^ = 1.0 Hz, 2H, Ar), 7.68 (dd, J^1^ = 8.5 Hz, J^2^ = 1.0 Hz, 2H, Ar). ^13^C NMR [CDCl_3_, 125 MHz, δ (p.p.m.)]: 28.2, 34.5, 41.0, 72.5, 73.4, 77.8, 125.3, 125.4, 126.1, 126.2, 126.9, 127.2, 128.0, 128.1, 128.4, 142.6, 146.6, 148.0. IR (film, cm^-1^): 3428, 3263, 1449. MS (ESI): 209, 167. HRMS (ESI) calc for C_24_H_25_NO + H: 344.2014, found: 344.2083.

### Refinement

All H-atoms were placed in calculated positions (O—H = 0.84 Å, and C—H 0.95 to 1.00 Å) and were included in the refinement in the riding model approximation with *U_iso_*(H) = 1.2*U_eq_*(C) and 1.5*U*_eq_(O; methyl-C). In the absence of significant anomalous scattering effects, 1707 Friedel pairs were averaged in the final refinement. The 2*S*,5*R* designation was chosen based on the synthesis (Moro *et al.*, 2010). The C7–C12 benzene ring was found to be disordered with one orientation slightly displaced with respect to the second, co-planar, orientation. In the final refinement, matching C atoms were constrained to have the same anisotropic displacement parameter. The major component of the disordered residue had a site occupancy factor = 0.782 (15).

### Crystal data

**Table:**

C_24_H_25_NO	*F*(000) = 736
*M**_r_* = 343.45	*D*_x_ = 1.189 Mg m^−^^3^
Orthorhombic, *P*2_1_2_1_2_1_	Mo *K*α radiation, λ = 0.71073 Å
Hall symbol: P 2ac 2ab	Cell parameters from 7679 reflections
*a* = 9.9672 (2) Å	θ = 2.5–25.5°
*b* = 13.3376 (2) Å	µ = 0.07 mm^−^^1^
*c* = 14.4369 (2) Å	*T* = 100 K
*V* = 1919.22 (5) Å^3^	Irregular, colourless
*Z* = 4	0.22 × 0.15 × 0.15 mm

### Data collection

**Table:**

Bruker APEXII CCD diffractometer	1979 reflections with *I* > 2σ(*I*)
Radiation source: fine-focus sealed tube	*R*_int_ = 0.039
graphite	θ_max_ = 26.5°, θ_min_ = 2.1°
φ and ω scans	*h* = −12→12
24972 measured reflections	*k* = −16→16
2262 independent reflections	*l* = −18→18

### Refinement

**Table:**

Refinement on *F*^2^	Secondary atom site location: difference Fourier map
Least-squares matrix: full	Hydrogen site location: inferred from neighbouring sites
*R*[*F*^2^ > 2σ(*F*^2^)] = 0.038	H-atom parameters constrained
*wR*(*F*^2^) = 0.092	*w* = 1/[σ^2^(*F*_o_^2^) + (0.0477*P*)^2^ + 0.2712*P*] where *P* = (*F*_o_^2^ + 2*F*_c_^2^)/3
*S* = 1.05	(Δ/σ)_max_ < 0.001
2262 reflections	Δρ_max_ = 0.17 e Å^−^^3^
255 parameters	Δρ_min_ = −0.19 e Å^−^^3^
0 restraints	Absolute structure: nd
Primary atom site location: structure-invariant direct methods	

### Special details

**Table:**

Geometry. All s.u.'s (except the s.u. in the dihedral angle between two l.s. planes) are estimated using the full covariance matrix. The cell s.u.'s are taken into account individually in the estimation of s.u.'s in distances, angles and torsion angles; correlations between s.u.'s in cell parameters are only used when they are defined by crystal symmetry. An approximate (isotropic) treatment of cell s.u.'s is used for estimating s.u.'s involving l.s. planes.
Refinement. Refinement of *F*^2^ against ALL reflections. The weighted *R*-factor *wR* and goodness of fit *S* are based on *F*^2^, conventional *R*-factors *R* are based on *F*, with *F* set to zero for negative *F*^2^. The threshold expression of *F*^2^ > 2σ(*F*^2^) is used only for calculating *R*-factors(gt) *etc*. and is not relevant to the choice of reflections for refinement. *R*-factors based on *F*^2^ are statistically about twice as large as those based on *F*, and *R*- factors based on ALL data will be even larger.

### Fractional atomic coordinates and isotropic or equivalent isotropic displacement parameters (Å^2^)

**Table:**

	*x*	*y*	*z*	*U*_iso_*/*U*_eq_	Occ. (<1)
O	0.11866 (14)	0.86869 (11)	0.21128 (12)	0.0486 (4)	
H1o	0.1387	0.8166	0.1816	0.073*	
N	0.30995 (17)	0.77434 (11)	0.11848 (11)	0.0360 (4)	
C1	0.3091 (2)	0.74937 (15)	0.01898 (13)	0.0364 (4)	
H1	0.4023	0.7558	−0.0058	0.044*	
C2	0.2230 (2)	0.83278 (15)	−0.02138 (14)	0.0422 (5)	
H2A	0.2437	0.8432	−0.0877	0.051*	
H2B	0.1264	0.8169	−0.0149	0.051*	
C3	0.2595 (2)	0.92449 (15)	0.03493 (14)	0.0409 (5)	
H3A	0.1786	0.9652	0.0481	0.049*	
H3B	0.3250	0.9665	0.0010	0.049*	
C4	0.3212 (2)	0.88486 (13)	0.12565 (12)	0.0327 (4)	
H4	0.4177	0.9050	0.1300	0.039*	
C5	0.4120 (2)	0.71912 (17)	0.16989 (15)	0.0510 (6)	
H5A	0.3976	0.6470	0.1619	0.076*	
H5B	0.4059	0.7362	0.2358	0.076*	
H5C	0.5011	0.7371	0.1465	0.076*	
C12	0.24357 (19)	0.92007 (13)	0.21337 (13)	0.0333 (4)	
C25	0.2101 (7)	1.0299 (5)	0.2126 (5)	0.0299 (10)	0.782 (15)
C26	0.0798 (6)	1.0643 (6)	0.2313 (5)	0.0443 (11)	0.782 (15)
H26	0.0112	1.0168	0.2439	0.053*	0.782 (15)
C27	0.0485 (7)	1.1642 (4)	0.2320 (3)	0.0504 (12)	0.782 (15)
H27	−0.0405	1.1847	0.2458	0.060*	0.782 (15)
C28	0.1454 (8)	1.2356 (3)	0.2127 (3)	0.0462 (14)	0.782 (15)
H28	0.1232	1.3049	0.2128	0.055*	0.782 (15)
C29	0.2743 (7)	1.2049 (4)	0.1933 (3)	0.0441 (15)	0.782 (15)
H29	0.3421	1.2532	0.1810	0.053*	0.782 (15)
C30	0.3055 (7)	1.1029 (6)	0.1918 (5)	0.0383 (11)	0.782 (15)
H30	0.3940	1.0827	0.1762	0.046*	0.782 (15)
C13	0.3186 (2)	0.89712 (14)	0.30368 (13)	0.0396 (5)	
C14	0.2559 (3)	0.84209 (16)	0.37346 (15)	0.0563 (7)	
H14	0.1668	0.8185	0.3648	0.068*	
C15	0.3229 (5)	0.8218 (2)	0.45513 (17)	0.0781 (10)	
H15	0.2799	0.7831	0.5017	0.094*	
C16	0.4509 (4)	0.8566 (2)	0.46997 (18)	0.0800 (11)	
H16	0.4959	0.8426	0.5265	0.096*	
C17	0.5129 (3)	0.91177 (18)	0.40224 (18)	0.0656 (8)	
H17	0.6011	0.9366	0.4121	0.079*	
C18	0.4475 (2)	0.93152 (15)	0.31956 (16)	0.0479 (5)	
H18	0.4920	0.9693	0.2730	0.057*	
C6	0.2582 (2)	0.64512 (15)	−0.00095 (14)	0.0399 (5)	
C7	0.3065 (3)	0.59257 (18)	−0.07668 (16)	0.0548 (6)	
H7	0.3759	0.6207	−0.1136	0.066*	
C8	0.2548 (4)	0.4995 (2)	−0.0990 (2)	0.0711 (8)	
H8	0.2877	0.4650	−0.1519	0.085*	
C9	0.1568 (3)	0.45654 (19)	−0.0460 (2)	0.0705 (8)	
H9	0.1223	0.3923	−0.0616	0.085*	
C10	0.1091 (3)	0.50665 (18)	0.0295 (2)	0.0645 (7)	
H10	0.0417	0.4768	0.0670	0.077*	
C11	0.1583 (3)	0.60091 (17)	0.05168 (18)	0.0544 (6)	
H11	0.1230	0.6356	0.1037	0.065*	
C19	0.235 (3)	1.043 (2)	0.204 (2)	0.0299 (10)	0.218 (15)
C20	0.116 (2)	1.072 (3)	0.238 (2)	0.0443 (11)	0.218 (15)
H20	0.0495	1.0280	0.2623	0.053*	0.218 (15)
C21	0.101 (3)	1.1852 (17)	0.2343 (15)	0.0504 (12)	0.218 (15)
H21	0.0187	1.2167	0.2509	0.060*	0.218 (15)
C22	0.207 (3)	1.2397 (15)	0.2064 (13)	0.0462 (14)	0.218 (15)
H22	0.1991	1.3107	0.2057	0.055*	0.218 (15)
C23	0.323 (2)	1.1984 (16)	0.1794 (14)	0.0441 (15)	0.218 (15)
H23	0.3949	1.2404	0.1602	0.053*	0.218 (15)
C24	0.340 (2)	1.098 (2)	0.179 (2)	0.0383 (11)	0.218 (15)
H24	0.4225	1.0679	0.1620	0.046*	0.218 (15)

### Atomic displacement parameters (Å^2^)

**Table:**

	*U*^11^	*U*^22^	*U*^33^	*U*^12^	*U*^13^	*U*^23^
O	0.0386 (8)	0.0402 (8)	0.0671 (10)	−0.0100 (7)	0.0144 (7)	−0.0061 (7)
N	0.0446 (9)	0.0298 (8)	0.0337 (8)	0.0063 (8)	−0.0033 (8)	−0.0016 (6)
C1	0.0357 (10)	0.0407 (10)	0.0327 (10)	0.0019 (9)	0.0008 (8)	−0.0048 (8)
C2	0.0501 (12)	0.0411 (11)	0.0355 (10)	−0.0006 (10)	−0.0064 (9)	0.0013 (9)
C3	0.0459 (12)	0.0377 (10)	0.0392 (11)	−0.0011 (10)	−0.0040 (9)	0.0025 (8)
C4	0.0331 (10)	0.0304 (9)	0.0344 (9)	−0.0005 (8)	−0.0009 (8)	0.0012 (7)
C5	0.0668 (15)	0.0439 (12)	0.0423 (12)	0.0218 (11)	−0.0111 (11)	−0.0075 (9)
C12	0.0318 (9)	0.0277 (9)	0.0405 (10)	−0.0023 (8)	0.0049 (8)	−0.0005 (8)
C25	0.034 (3)	0.026 (2)	0.0295 (18)	−0.0063 (17)	0.0048 (17)	0.0031 (14)
C26	0.042 (3)	0.0436 (18)	0.047 (2)	0.008 (3)	0.005 (3)	−0.0021 (14)
C27	0.055 (3)	0.042 (2)	0.0541 (15)	0.013 (2)	0.011 (2)	−0.0058 (15)
C28	0.068 (4)	0.0304 (12)	0.0402 (14)	0.012 (2)	0.002 (2)	−0.0049 (11)
C29	0.059 (4)	0.0318 (14)	0.042 (2)	−0.005 (3)	−0.006 (2)	0.0003 (14)
C30	0.035 (3)	0.0340 (13)	0.046 (2)	−0.003 (2)	−0.002 (2)	0.0041 (16)
C13	0.0591 (13)	0.0253 (9)	0.0344 (10)	0.0074 (9)	0.0060 (9)	−0.0043 (7)
C14	0.0913 (18)	0.0380 (11)	0.0395 (12)	0.0125 (13)	0.0202 (12)	0.0008 (9)
C15	0.151 (3)	0.0489 (15)	0.0343 (13)	0.030 (2)	0.0228 (17)	0.0036 (11)
C16	0.149 (3)	0.0556 (16)	0.0352 (13)	0.042 (2)	−0.0182 (17)	−0.0086 (12)
C17	0.091 (2)	0.0513 (13)	0.0544 (15)	0.0220 (15)	−0.0274 (14)	−0.0158 (12)
C18	0.0645 (14)	0.0357 (11)	0.0434 (12)	0.0060 (11)	−0.0100 (11)	−0.0020 (9)
C6	0.0434 (11)	0.0362 (10)	0.0401 (11)	0.0066 (9)	−0.0071 (9)	−0.0035 (8)
C7	0.0678 (15)	0.0495 (13)	0.0473 (12)	0.0054 (13)	−0.0030 (12)	−0.0123 (10)
C8	0.090 (2)	0.0554 (14)	0.0674 (17)	0.0117 (15)	−0.0144 (17)	−0.0271 (13)
C9	0.0798 (19)	0.0372 (12)	0.094 (2)	0.0030 (13)	−0.0359 (18)	−0.0136 (14)
C10	0.0615 (15)	0.0442 (13)	0.088 (2)	−0.0066 (12)	−0.0111 (15)	0.0042 (14)
C11	0.0562 (14)	0.0433 (12)	0.0637 (15)	−0.0025 (11)	0.0020 (12)	−0.0047 (11)
C19	0.034 (3)	0.026 (2)	0.0295 (18)	−0.0063 (17)	0.0048 (17)	0.0031 (14)
C20	0.042 (3)	0.0436 (18)	0.047 (2)	0.008 (3)	0.005 (3)	−0.0021 (14)
C21	0.055 (3)	0.042 (2)	0.0541 (15)	0.013 (2)	0.011 (2)	−0.0058 (15)
C22	0.068 (4)	0.0304 (12)	0.0402 (14)	0.012 (2)	0.002 (2)	−0.0049 (11)
C23	0.059 (4)	0.0318 (14)	0.042 (2)	−0.005 (3)	−0.006 (2)	0.0003 (14)
C24	0.035 (3)	0.0340 (13)	0.046 (2)	−0.003 (2)	−0.002 (2)	0.0041 (16)

### Geometric parameters (Å, °)

**Table:**

O—C12	1.421 (2)	C13—C18	1.383 (3)
O—H1o	0.8401	C13—C14	1.394 (3)
N—C5	1.459 (3)	C14—C15	1.382 (4)
N—C1	1.475 (2)	C14—H14	0.9500
N—C4	1.482 (2)	C15—C16	1.374 (5)
C1—C6	1.508 (3)	C15—H15	0.9500
C1—C2	1.521 (3)	C16—C17	1.371 (4)
C1—H1	1.0000	C16—H16	0.9500
C2—C3	1.513 (3)	C17—C18	1.385 (3)
C2—H2A	0.9900	C17—H17	0.9500
C2—H2B	0.9900	C18—H18	0.9500
C3—C4	1.541 (3)	C6—C11	1.384 (3)
C3—H3A	0.9900	C6—C7	1.385 (3)
C3—H3B	0.9900	C7—C8	1.383 (4)
C4—C12	1.557 (3)	C7—H7	0.9500
C4—H4	1.0000	C8—C9	1.366 (5)
C5—H5A	0.9800	C8—H8	0.9500
C5—H5B	0.9800	C9—C10	1.364 (4)
C5—H5C	0.9800	C9—H9	0.9500
C12—C25	1.503 (7)	C10—C11	1.387 (3)
C12—C13	1.534 (3)	C10—H10	0.9500
C12—C19	1.65 (3)	C11—H11	0.9500
C25—C30	1.394 (7)	C19—C24	1.32 (3)
C25—C26	1.403 (9)	C19—C20	1.34 (4)
C26—C27	1.368 (9)	C20—C21	1.51 (4)
C26—H26	0.9500	C20—H20	0.9500
C27—C28	1.385 (6)	C21—C22	1.34 (2)
C27—H27	0.9500	C21—H21	0.9500
C28—C29	1.378 (5)	C22—C23	1.34 (2)
C28—H28	0.9500	C22—H22	0.9500
C29—C30	1.395 (9)	C23—C24	1.35 (4)
C29—H29	0.9500	C23—H23	0.9500
C30—H30	0.9500	C24—H24	0.9500
			
C12—O—H1o	101.6	C25—C30—C29	121.7 (5)
C5—N—C1	112.69 (16)	C25—C30—H30	119.1
C5—N—C4	114.43 (16)	C29—C30—H30	119.1
C1—N—C4	107.05 (14)	C18—C13—C14	118.1 (2)
N—C1—C6	113.34 (16)	C18—C13—C12	121.84 (18)
N—C1—C2	102.20 (16)	C14—C13—C12	120.0 (2)
C6—C1—C2	114.29 (17)	C15—C14—C13	120.2 (3)
N—C1—H1	108.9	C15—C14—H14	119.9
C6—C1—H1	108.9	C13—C14—H14	119.9
C2—C1—H1	108.9	C16—C15—C14	121.0 (3)
C3—C2—C1	104.46 (16)	C16—C15—H15	119.5
C3—C2—H2A	110.9	C14—C15—H15	119.5
C1—C2—H2A	110.9	C17—C16—C15	119.3 (3)
C3—C2—H2B	110.9	C17—C16—H16	120.4
C1—C2—H2B	110.9	C15—C16—H16	120.4
H2A—C2—H2B	108.9	C16—C17—C18	120.3 (3)
C2—C3—C4	105.99 (16)	C16—C17—H17	119.8
C2—C3—H3A	110.5	C18—C17—H17	119.8
C4—C3—H3A	110.5	C13—C18—C17	121.1 (2)
C2—C3—H3B	110.5	C13—C18—H18	119.5
C4—C3—H3B	110.5	C17—C18—H18	119.5
H3A—C3—H3B	108.7	C11—C6—C7	117.9 (2)
N—C4—C3	104.56 (15)	C11—C6—C1	122.02 (19)
N—C4—C12	108.61 (15)	C7—C6—C1	120.0 (2)
C3—C4—C12	112.90 (15)	C8—C7—C6	120.6 (3)
N—C4—H4	110.2	C8—C7—H7	119.7
C3—C4—H4	110.2	C6—C7—H7	119.7
C12—C4—H4	110.2	C9—C8—C7	120.8 (3)
N—C5—H5A	109.5	C9—C8—H8	119.6
N—C5—H5B	109.5	C7—C8—H8	119.6
H5A—C5—H5B	109.5	C10—C9—C8	119.4 (3)
N—C5—H5C	109.5	C10—C9—H9	120.3
H5A—C5—H5C	109.5	C8—C9—H9	120.3
H5B—C5—H5C	109.5	C9—C10—C11	120.3 (3)
O—C12—C25	106.0 (3)	C9—C10—H10	119.8
O—C12—C13	110.43 (16)	C11—C10—H10	119.8
C25—C12—C13	108.0 (3)	C6—C11—C10	120.9 (2)
O—C12—C4	105.84 (15)	C6—C11—H11	119.5
C25—C12—C4	113.5 (3)	C10—C11—H11	119.5
C13—C12—C4	112.86 (15)	C24—C19—C20	130 (3)
O—C12—C19	115.5 (11)	C24—C19—C12	122 (2)
C13—C12—C19	107.2 (12)	C20—C19—C12	108 (2)
C4—C12—C19	105.0 (10)	C19—C20—C21	111 (2)
C30—C25—C26	116.4 (6)	C19—C20—H20	124.5
C30—C25—C12	122.1 (5)	C21—C20—H20	124.5
C26—C25—C12	121.5 (5)	C22—C21—C20	118.2 (18)
C27—C26—C25	122.1 (5)	C22—C21—H21	120.9
C27—C26—H26	119.0	C20—C21—H21	120.9
C25—C26—H26	119.0	C23—C22—C21	123.0 (18)
C26—C27—C28	120.6 (4)	C23—C22—H22	118.5
C26—C27—H27	119.7	C21—C22—H22	118.5
C28—C27—H27	119.7	C22—C23—C24	121 (2)
C29—C28—C27	119.1 (4)	C22—C23—H23	119.5
C29—C28—H28	120.4	C24—C23—H23	119.5
C27—C28—H28	120.4	C19—C24—C23	117 (2)
C28—C29—C30	120.0 (4)	C19—C24—H24	121.7
C28—C29—H29	120.0	C23—C24—H24	121.7
C30—C29—H29	120.0		
			
C5—N—C1—C6	70.2 (2)	O—C12—C13—C14	−6.8 (2)
C4—N—C1—C6	−163.11 (17)	C25—C12—C13—C14	108.7 (3)
C5—N—C1—C2	−166.29 (18)	C4—C12—C13—C14	−124.99 (18)
C4—N—C1—C2	−39.6 (2)	C19—C12—C13—C14	119.8 (10)
N—C1—C2—C3	36.6 (2)	C18—C13—C14—C15	−1.0 (3)
C6—C1—C2—C3	159.41 (17)	C12—C13—C14—C15	−179.62 (19)
C1—C2—C3—C4	−20.7 (2)	C13—C14—C15—C16	1.2 (4)
C5—N—C4—C3	152.32 (17)	C14—C15—C16—C17	−0.4 (4)
C1—N—C4—C3	26.7 (2)	C15—C16—C17—C18	−0.4 (4)
C5—N—C4—C12	−86.9 (2)	C14—C13—C18—C17	0.2 (3)
C1—N—C4—C12	147.48 (16)	C12—C13—C18—C17	178.72 (19)
C2—C3—C4—N	−3.0 (2)	C16—C17—C18—C13	0.6 (3)
C2—C3—C4—C12	−120.85 (18)	N—C1—C6—C11	34.1 (3)
N—C4—C12—O	−45.89 (19)	C2—C1—C6—C11	−82.4 (2)
C3—C4—C12—O	69.60 (19)	N—C1—C6—C7	−149.2 (2)
N—C4—C12—C25	−161.7 (3)	C2—C1—C6—C7	94.2 (2)
C3—C4—C12—C25	−46.2 (4)	C11—C6—C7—C8	0.8 (3)
N—C4—C12—C13	75.00 (19)	C1—C6—C7—C8	−176.0 (2)
C3—C4—C12—C13	−169.51 (16)	C6—C7—C8—C9	−1.3 (4)
N—C4—C12—C19	−168.5 (12)	C7—C8—C9—C10	0.5 (4)
C3—C4—C12—C19	−53.1 (12)	C8—C9—C10—C11	0.7 (4)
O—C12—C25—C30	−164.1 (6)	C7—C6—C11—C10	0.4 (3)
C13—C12—C25—C30	77.5 (7)	C1—C6—C11—C10	177.2 (2)
C4—C12—C25—C30	−48.4 (8)	C9—C10—C11—C6	−1.2 (4)
C19—C12—C25—C30	−10 (7)	O—C12—C19—C24	−161 (3)
O—C12—C25—C26	14.7 (7)	C25—C12—C19—C24	172 (10)
C13—C12—C25—C26	−103.7 (7)	C13—C12—C19—C24	76 (3)
C4—C12—C25—C26	130.4 (6)	C4—C12—C19—C24	−45 (3)
C19—C12—C25—C26	169 (8)	O—C12—C19—C20	28 (3)
C30—C25—C26—C27	−2.0 (11)	C25—C12—C19—C20	1(6)
C12—C25—C26—C27	179.2 (6)	C13—C12—C19—C20	−95 (2)
C25—C26—C27—C28	0.9 (10)	C4—C12—C19—C20	144 (2)
C26—C27—C28—C29	−0.4 (7)	C24—C19—C20—C21	9(5)
C27—C28—C29—C30	1.0 (7)	C12—C19—C20—C21	179 (2)
C26—C25—C30—C29	2.6 (11)	C19—C20—C21—C22	−6(4)
C12—C25—C30—C29	−178.5 (5)	C20—C21—C22—C23	2(4)
C28—C29—C30—C25	−2.2 (9)	C21—C22—C23—C24	0(4)
O—C12—C13—C18	174.73 (17)	C20—C19—C24—C23	−7(5)
C25—C12—C13—C18	−69.8 (3)	C12—C19—C24—C23	−176 (2)
C4—C12—C13—C18	56.5 (2)	C22—C23—C24—C19	2(4)
C19—C12—C13—C18	−58.7 (10)		

### Hydrogen-bond geometry (Å, °)

**Table:**

Cg is the centroid of the C6–C11 ring.

**Table:**

*D*—H···*A*	*D*—H	H···*A*	*D*···*A*	*D*—H···*A*
O—H1O···N	0.84	2.02	2.648 (2)	132
C28—H28···O^i^	0.95	2.78	3.359 (7)	120
C17—H17···Cg^ii^	0.95	2.92	3.776 (3)	150

Symmetry codes: (i) −*x*, *y*+1/2, −*z*+1/2; (ii) −*x*+1, *y*−1/2, −*z*+1/2.
